# Single-Mode Lasing in Polymer Circular Gratings

**DOI:** 10.3390/ma14092318

**Published:** 2021-04-29

**Authors:** Saisai Chu, Anwer Hayat, Fengzhao Cao, Tianrui Zhai

**Affiliations:** 1 State Key Laboratory for Mesoscopic Physics, Department of Physics, Peking University, Beijing 100871, China; chusaisai@pku.edu.cn; 2 Institute of Information Photonics Technology, College of Applied Sciences, Beijing University of Technology, Beijing 100124, China; anwerhayatnoor@gmail.com (A.H.); wincfz@emails.bjut.edu.cn (F.C.)

**Keywords:** circular gratings, single-mode lasing, polymer lasers

## Abstract

In recent years, conjugated polymers have become the materials of choice to fabricate optoelectronic devices, owing to their properties of high absorbance, high quantum efficiency, and wide luminescence tuning ranges. The efficient feedback mechanism in the concentric ring resonator and its circularly symmetric periodic geometry combined with the broadband photoluminescence spectrum of the conjugated polymer can generate a highly coherent output beam. Here, the detailed design of the ultralow-threshold single-mode circular distributed feedback polymer laser is presented with combined fabrication processes such as electron beam lithography and the spin-coating technique. We observe from the extinction spectra of the circular gratings that the transverse electric mode shows no change with the increase of incident beam angle. The strong enhancement of the conjugated polymer photoluminescence spectra with the circular periodic resonator can reduce the lasing threshold about 19 µJ/cm^2^. A very thin polymer film of about 110 nm is achieved with the spin-coating technique. The thickness of the gain medium can support only the zero-order transverse electric lasing mode. We expect that such a low threshold lasing device can find application in optoelectronic devices.

## 1. Introduction

In optoelectronic and also in laser technology, gain materials are regarded as the building block that can improve device performance, and some of them open a new route towards real-life applications. Similarly, common gain materials, such as inorganic semiconductors, organic dyes, organic semiconductors, quantum dots, and perovskites, are used in laser systems with small differences in thin film fabrication properties, electrical conductivity, and difficulties in the manufacturing process [1,2,3,4,5,6,7,8,9]. Among them, organic semiconductors are composed of different element of chromophores with conjugated fragments. In organic semiconductors, conjugated polymers have magnificent optical and electrical properties, broad photoluminescence (PL) spectral in the visible region, lost-cost processing, and easy fabrication methods on the flexible substrates [3,10,11,12,13]. Therefore, all these unique and intriguing features of the conjugated polymers provide a new route to explore optoelectronic technologies and micro-cavity lasers [14,15]. In recent years, conjugated polymers have been extensively applied as gain mediums in laser systems with different optical feedback nanostructures, such as whispering gallery mode (WGM)[16,17], micro-droplet [18], and distributed feedback (DFB) cavities [19,20,21,22,23]. Modern research considers many conjugated polymers in lasing applications, such as poly [9, 9-dioctylfluorenyl-2,7-diyl]–end capped with DMP (PFO), poly[(9,9-dioctylfluorenyl-2,7-diyl)-alt-co-(1,4-benzo-(2,1′,3) -thiadiazole)] (F8BT), and poly[2 -methoxy-5-(3′,7′-dimethyloctyloxy)-1,4-phenylenevinylene] (MDMO-PPV) covering the whole visible spectra, which can generate coherent light either pump optically or electrically [24,25,26].

Recently, various strategies have been proposed to engineer the operating characteristic of the second-order and high-order DFB polymer lasers, especially to control the output-coupled lasing beam (surface-emitting) with low divergence angle along and normal to the periodic nanostructures [27,28,29]. The performance of the DFB polymer lasers can be further improved by reducing the lasing threshold by manipulating the photonic mode density. Similarly, three-dimensional (3D) photonic band gap structures can be regarded as ideal candidates for attaining a low threshold by confining the photonic mode but the fabrication process and modification in structure are complicated. This problem can be resolved by adopting the circular grating structure already known from the literature [30,31,32,33,34]; the feedback mechanism in the cavity is similar to the second-order DFB lasers [35]. The circular periodic structures provide a nearly 2D feedback mechanism via second-order Bragg reflection process in the grating plane and simultaneously emit vertical radiation through first-order Bragg diffraction [36]. In circular periodic structures, in-plane momentum is conserved for radial directions and wave-guided mode leads to constructive interference of radiation out of the plane via Bragg scatterings. The Bragg diffraction process for the second-order DFB circular grating structures (CGS) can be written as [37]:

(1) $$k_{e}\sin\beta =\pm k_{g}\pm nG=\pm \frac{2\pi n_{eff}}{\lambda_{e}}\pm \frac{2\pi n}{\Lambda }$$

In the above equation, $k_{e}$ and $k_{g}$ are the emitted and guided-mode radiation wave vectors, whereas G and β are the Bragg grating vector and output coupling angle (angle of emission) related to the guided mode wave vector. The comprehensive section of the Equation (1) includes $n_{eff}$ (effective refractive index), $\lambda_{e}$ (emission wavelength), Λ (circular grating period), and *n* (integer for grating order), respectively. Therefore, surface-emitting lasing from the circular grating structure is obtained via first-order diffraction (*β* = 0) and the above equation can be further modified as; $\lambda_{e}{=n}_{eff}\;\Lambda$. Similarly, the 2D feedback mechanism in the circular grating structures combine with the broadband emission spectrum of the conjugated polymer would be an excellent candidate for attaining high-performance, low-threshold, single-mode lasers with high Q factor in the visible region. To our knowledge, second-order circular grating structures with ultra-thin film of the conjugated polymer showing low threshold, ${\mathrm{TE}}_{0}$ mode, and single-mode lasing have not been reported yet.

In this article, we focus on the morphology and lasing performance of optically pumped surface-emitting circular DFB polymer laser. The solution of PMMA in chlorobenzene was spin-coated on a glass (BK7) substrate, and after the baking process circular grating structures were written with electron beam lithography (EBL). The gain material conjugated polymer F8BT was spin-coated on the prepared circular grating structures. Finally, the circular DFB polymer laser was optically pumped to investigate the lasing properties such as lasing thresholds and emission wavelength. 

## 2. Fabrication Methods and Materials

**Figure 1:**
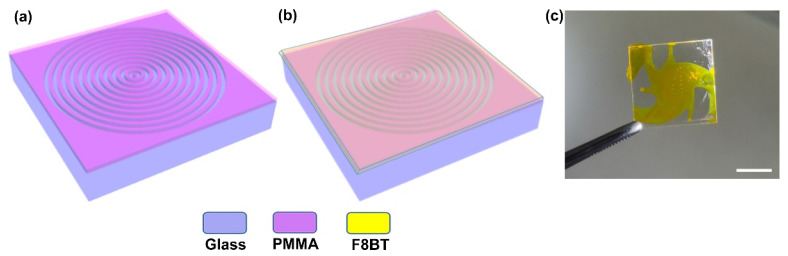
Schematic layout of the fabrication process. (**a**) The schematic layout of the circular periodic nanostructures written with the electron beam lithography (EBL). The blue-violet and purple color showing the glass substrate and electron beam resist PMMA film, respectively. (**b**) The schematic illustration of the gain material F8BT on the circular periodic geometry. The yellow color defines a gain medium F8BT film in the schematic layout. (**c**) The photograph of the actual composite; the scale bar is 1 cm.

In this work, we applied electron beam lithography, a simple and effective fabrication technique for writing the geometry of circular periodic structures on the glass substrate, which would provide essential feedback mechanism for attaining the surface-emitting circular DFB polymer laser. Figure 1 illustrates the whole schematic diagram of the optically pumped circular grating DFB polymer lasing device used in our experiment. Initially, a solution of electron-beam resist polymethylmethacrylate (PMMA) with a concentration of 4.0% in chlorobenzene was spin-coated on a glass substrate (10 mm × 10 mm × 1mm). Similarly, we have achieved a PMMA film having a thickness of 160 nm at a spin-coating speed of 4000 rpm for 30 s. The prepared film of the electron-beam resist (PMMA) was baked on a hot plate at 150 °C for 2 min. The same spin-coating technique was utilized to cover the electron beam resist film with 20 nm an anti-charging (AR-PC 5090.02, Allresist GmbH, Strausberg, Germany) layer. Hence, the electron beam lithography (Raith e_LiNE plus, Brisbane, Australia) with an accelerating voltage of 30 kV and working distance of 10 mm was utilized to write 2D circular periodic structures on the developed sample. After that, the sample was rinsed with DI water for removing the anti-charge layer and then developed with MIBK: IPA (1:3) and stopped with IPA for 30 s, respectively. The schematic of the circular periodic nanostructures after the whole process is illustrated in the Figure 1a. Here, we used a conjugated polymer F8BT as a gain material, whose gain peak can be realized around 570 nm wavelength [38]. The gain material F8BT was dissolved in the xylene at a concentration of 23.5 mg/mL. The solution of the F8BT was spin-coated on circular grating periodic nanostructures at 1500 rpm for 30 s. At this spin-coating speed, we realized approximately 110 nm thickened film of the F8BT on the circular grating structures. The final form of the circular grating DFB lasing device is schematically depicted in the Figure 1b. The actual composite photograph is shown in Figure 1c.

## 3. Experiment Results

**Figure 2:**
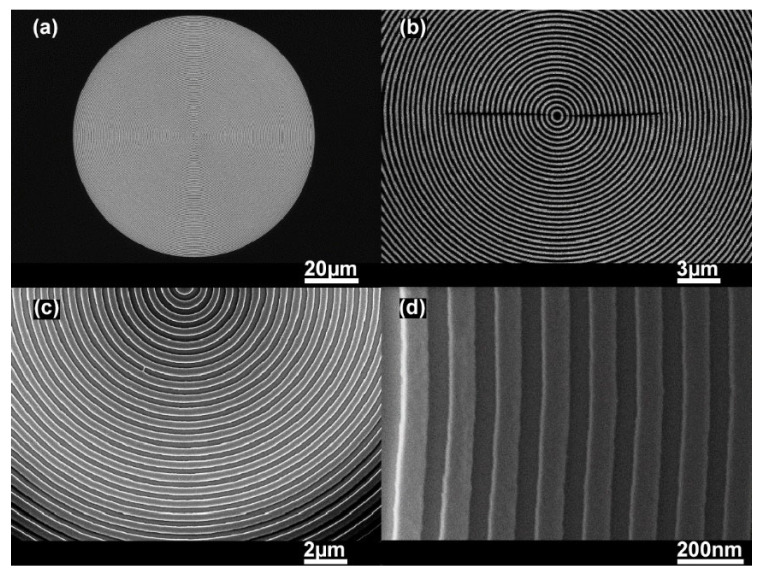
Scanning electron microscopic (SEM) images of the circular periodic nanostructures at (**a**) 1.03 k×, (**b**) 6.74 k×, (**c**) 12.29 k×, and (**d**) 42.08 k× magnifications, respectively, showing the well-defined morphology of the respective ring cavities.

Improved insight into the developed circular grating DFB polymer laser system can be gained by investigating its various properties such as morphology of the circular grating nanostructures, optical microscopic images, optical spectroscopic characterization, and lasing properties. The morphology of the circular grating structure was studied through the scanning electron microscope (SEM) to check the quality of the feedback cavity. The SEM photographs of the circular periodic nanostructures on the glass substrate at various magnification powers are depicted in the Figure 2a–d. As we can observe from the SEM photographs, the circular periodic nanostructures from high to low magnification power are free of defects and have considerable equal spacing between each modulation depth of the circular ring, indicating a significant interest in high-quality laser systems. In our experiment, the period of the circular grating was about 350 nm with respect to the second-order Brag equation used for DFB lasers.

**Figure 3:**
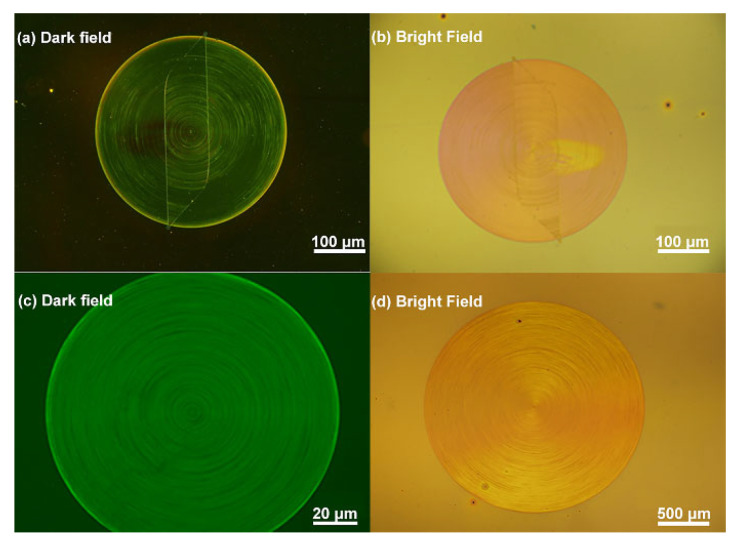
Optical microscopic images of the circular periodic structures. (**a**) and (**c**): Dark-field optical micrographs. (**b**) and (**d**): Bright-field optical micrographs.

Figure 3 displays dark and bright field optical micrograph of the circular periodic nanostructures at different magnification powers. The circular periodic geometry written with the EBL is highly uniform across the whole area of the gratings and shows high fidelity down to the sub-wavelength level, as can be observed from both the dark- and bright-field optical micrographs (Figure 3).

**Figure 4:**
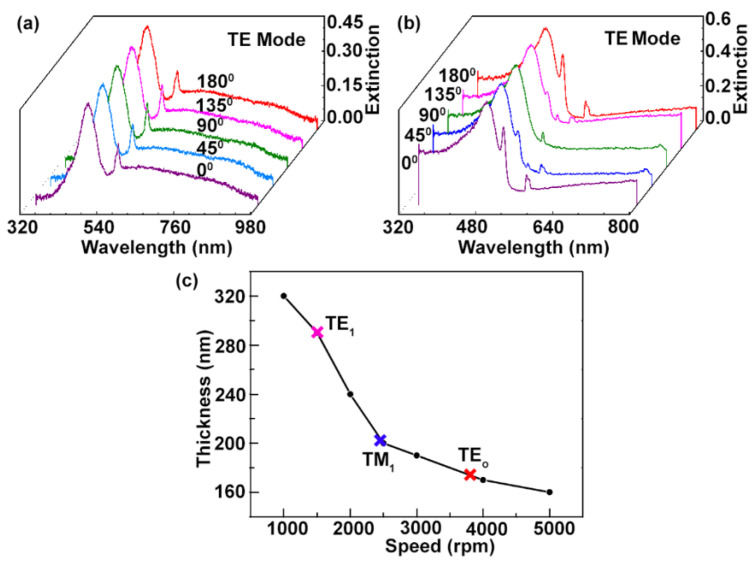
Optical characterization of the circular periodic grating and regular periodic grating. (**a**) Extension spectra of the circular grating at different polarization angle for ${\mathrm{TE}}_{0}$ mode. (**b**) Regular grating extinction curve indicating polarization at various angles for ${\mathrm{TE}}_{0}$ mode. (**c**) Thickness of the F8BT film as a function of spin-coating speed. The red, blue, and pink colors in the F8BT thickness curve represent ${\mathrm{TE}}_{0}$,${\mathrm{TM}}_{1}$ and ${\mathrm{TE}}_{1}$ mode availability, according to the variation of the film thickness.

In our experiment, we spin-coated a thin film of F8BT on the circular and regular (1D) grating structures to study the angle resolved polarization properties of the TE mode. Both the samples were excited with a non-polarized white light from tungsten halogen lamp (HL-2000, Ocean Optics, FL, USA). The optical transmission spectra were measured by using fiber spectrum analyzer from optical spectrometer (USB 4000, Ocean Optics, FL, USA). The incident white light can be TE polarized, when its electric field is parallel to the incident grating planes. Extinction spectrum for both the circular and regular gratings (one dimension, 1D) covered with a gain material F8BT can be achieved from the formula $-\log(I_{t}/I_{o})$, where $I_{t}$ and $I_{o}$ indicate the intensities of the transmitted spectrum, as shown in the curves of Figure 4a,b. The wide peaks in both the Figure 4a,b correspond to absorption spectrum of the conjugated polymer F8BT, whereas the narrow peaks show the coupling between the Bragg diffraction from the periodic structures (circular and regular) and waveguide (F8BT film) mode [23,39]. From the Figure 4a, it can be seen that by increasing the angle with a step of 45° there is no change in the extinction curves. These phenomena are observed due to the symmetric distribution of the periodic structures and efficient coupling between the circular concentric rings and waveguide mode, while Figure 4b represents the change in the extinction spectra by increasing the incident angle for the 1D regular grating geometry. A narrow peak of the extinction graph (in Figure 4b) would represent maximum intensity when the regular grating structure plane and incident white light electric field are parallel (θ = 0° and 180°) to each other. Therefore, the circular periodic geometry combined with the significant optical properties of the conjugated polymer F8BT as a gain medium can improve the lasing performance of the DFB polymer lasers.

In our experiment, we studied the single as well as ${\mathrm{TE}}_{0}$ lasing mode emitted from the circular grating polymer laser. For this purpose, we spin-coated F8BT on a glass substrate at different spin-coating speeds. Therefore, the relationship between the various spin-coating speeds and the F8BT film thickness is depicted in the Figure 3c. The thickness of the waveguide plays an important role in the emission of lasing mode. Therefore, the necessary critical thickness $d_{e}$ (TE mode critical thickness) and $d_{m}$ (TM mode critical thickness) for the waveguide (F8BT film) can be calculated from the below equations [40]:

(2) $$d_{e}=\frac{(2p_{e}+1)\lambda }{4\sqrt{n_{wg}{}^{2}-1}},(p_{e}=0,1,2\ldots \ldots )$$

(3) $$d_{m}=\frac{p_{m}\lambda }{2\sqrt{n_{wg}{}^{2}-1}},\;(p_{m}=1,2,3\ldots \ldots )$$

In the above equations, $p_{e}$ and $p_{m}$ are the *p*th order of TE and TM modes. The other parameters such as λ and $n_{wg}$ are the emission wavelength and refractive index of the waveguide, respectively. Similarly, for a given experiment in which $n_{g}=1.8$ (refractive index of F8BT), λ=577 nm (emission wavelength), and $p_{e}=0$, the critical thickness was about 96 nm. Hence, above this thickness one ${\mathrm{TE}}_{0}$ mode exists in the F8BT waveguide. For ${\mathrm{TM}}_{1}$ and ${\mathrm{TE}}_{1}$, mode the critical thickness of the F8BT film would be greater than 192 nm and 289 nm, respectively. The red, blue, and pink cross in the Figure 3c indicates the F8BT film critical thickness range for ${\mathrm{TE}}_{0}$, ${\mathrm{TM}}_{1}$, and ${\mathrm{TE}}_{1}$.

**Figure 5:**
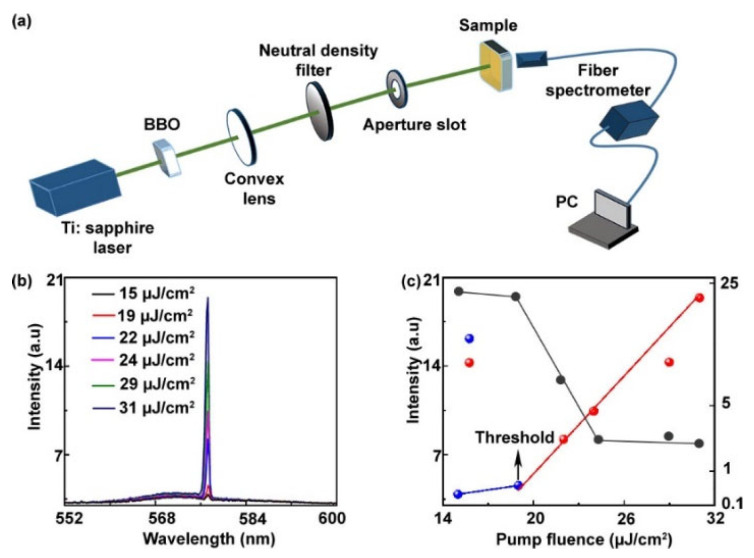
Lasing behavior of the circular periodic DFB polymer lasers. (**a**) Schematic optical layout out used for pumping lasing device. (**b**) Optical emission spectra at different pump fluences. (**c**) Evolution of the output intensity and the full width at half maxima as function of pump fluence. The black arrow in the graph indicates the threshold of the circular periodic DFB polymer laser about $19\;\mu J/{\mathrm{cm}}^{2}$

To underline the lasing emission properties, the measurements from the circular DFB polymer laser were carried out under optical pumping. Figure 5a illustrates the schematic of the optical layout used for the optical pumping. In this experiment, the prepared sample of circular grating polymer laser was excited with a frequency-doubled Ti:sapphire laser (400 nm wavelength, 1Khz repetition rate, 200 fs pulse duration). The optical pump beam after passing through the optical layout incident perpendicularly on the sample facing F8BT side toward the pump beam and the emission spectrum were recorded on the other side of the sample with fiber spectrometer (Maya 2000 Pro, Ocean Optics), as shown in Figure 5a. As we discussed at the beginning of the experiment, we designed our circular grating polymer laser according to the second-order DFB laser. Similarly, the single ${\mathrm{TE}}_{0}$ mode lasing was recorded at 577 nm, as illustrated in Figure 5b. When the excitation energy of the pump source is low (below the threshold, black line in the Figure 5b), very weak emission is recorded similar to the photoluminescence (PL) spectra. We detected a surface-emitting single- and zero-order TE modes with the increase of pump excitation energy above the threshold (see Figure 5b,c). Therefore, a sharp linear growth of the output emission intensity provided more insight into the lasing characteristics, as depicted in the Figure 5c. Lasing action can be observed in the circular grating DFB polymer laser with a minimum threshold of $19\;\mu J/{\mathrm{cm}}^{2}$, which is the lowest threshold as compared to previously reported circular grating DFB lasers DFB lasers [34,37]. In Figure 5c, the blue and red spherical balls define the intensity of emission spectra below and above the threshold, whereas the black arrow is the threshold point and the linear fit is indicated by a blue and red (below and above threshold) line for the guidance of the readers (see Figure 5c).

## 4. Conclusions

In summary, we have fabricated a single-mode circular grating DFB polymer laser by combining electron beam lithography and the spin-coating technique. The critical thickness for the zero-order TE mode was estimated with the formula given in the literature. Therefore, a convenient and easy fabrication (spin-coating) technique was applied to realize a very thin film of about 110 nm from the gain material F8BT. The optical extinction spectra section in our experiment can provide sufficient information about the efficient enhancement of the circular periodic geometry with the photoluminescence spectrum of the conjugated polymer F8BT. As a result, we have realized low-threshold (approximately $19\;\mu J/{\mathrm{cm}}^{2}$) single- and zero-order modes with a surface-emitting coherent beam in the circular grating DFB polymer laser. We expect that such a low threshold lasing device can find application in optoelectronic devices.

## Data Availability

Data sharing not available.
